# miR-21-5p Suppresses Mitophagy to Alleviate Hyperoxia-Induced Acute Lung Injury by Directly Targeting PGAM5

**DOI:** 10.1155/2020/4807254

**Published:** 2020-11-20

**Authors:** Guoyue Liu, Mingjiang Qian, Miao Chen, Tao Chen, Song Qin

**Affiliations:** ^1^The Second Affiliated Hospital of Zunyi Medical University, Zunyi, Guizhou 563000, China; ^2^Department of Critical Care Medicine, Affiliated Hospital of Zunyi Medical University, Zunyi, Guizhou 563000, China

## Abstract

Hyperoxia-induced acute lung injury (HALI) is a severe side effect of refractory hypoxemia treatment, for which no effective therapeutic strategy is available. Here, we found that the lung miR-21-5p level was significantly decreased in the rats subjected to hyperoxia. Further, we presented evidence that miR-21-5p was a crucial regulator of mitophagy and mitochondrial dysfunction. Moreover, it proved that miR-21-5p regulated hyperoxia-induced mitophagy and mitochondrial dysfunction by directly binding to the target gene PGAM5. In conclusion, for the first time, we found that miR-21-5p could directly suppress mitophagy and mitochondrial damage during HALI formation.

## 1. Introduction

Hyperoxia-induced acute lung injury (HALI) is a major clinical problem with high morbidity and mortality, which widely exists during the treatment of refractory hypoxemia [1]. It is reported that hyperoxia exposure stimulates the activation of inflammatory cells and thus results in pulmonary alveoli injury, alveolar-capillary leak, and the development of hyaline membranes [2, 3]. Unfortunately, no effective therapeutic strategies are available for HALI treatment. Therefore, it is necessary to understand further the mechanism of HALI to investigate novel pharmacotherapy.

Accumulated evidence found that mitochondrial dysfunction plays a central role in HALI [4, 5]. This dysfunction leads to the downregulation of ATP production, loss of mitochondrial membrane potential (*ΔΨ*m), and reactive oxygen species (ROS) accumulation in endothelial or epithelial cells. Ultimately, it contributes to mitochondria-mediated cell death, such as apoptosis, autophagy, and necrocytosis [6]. At present, much attention has been given to the events that HALI-induced mitochondrial dysfunction could trigger type II alveolar epithelial cell (AEC-II) apoptosis or necrocytosis [7–9]. However, in the context of HALI, few studies focus on the selective autophagy of mitochondria, termed as mitophagy. These findings result in a renewed interest in mitophagy and its role in HALI.

Nowadays, microRNAs (miRNAs) have been recently proved to be a new mediator of such stimuli, including ROS, mitochondrial dysfunction, and inflammation [10, 11]. It is a kind of small, endogenous, and noncoding RNA molecules with 21-25 nucleotides. They take part in various pathologic processes by directly targeting the essential genes' 3′ untranslated regions (3′UTR). In particular, Syed et al. had found that miR-34a was upregulated in the AEC-II cells and developing lungs, which were subjected to hyperoxia exposure. It suppressed the downstream target gene (angiopoietin-1) to induce the HALI and pulmonary arterial hypertension phenotype [3]. Similarly, in Prajapati et al.'s work, miRNAs have been confirmed to regulate neuron apoptosis by targeting transcript encoding subunit of mitochondrial complexes [12]. Thus, miRNAs have been considered a novel candidate for HALI therapy. However, whether miRNAs control mitophagy during HALI formation remains unclear. Of interest is miR-21-5p, which has been identified as a key mediator inducing acute lung injury and fibrosis [13, 14]. Intriguingly, our previous studies have reported that miR-21-5p was dramatically downregulated in the rat subjected to hyperoxic condition and inhibited apoptosis of type II alveolar epithelial cells through the PTEN/Akt pathway [2, 15]. However, whether miR-21-5p regulates mitophagy during HALI remains elusive.

For this purpose, we established a rat model to investigate the potential mechanism of HALI and found miR-21-5p dramatically decreased during this process, which might associate with HALI formation. Then, we investigated the function of miR-21-5p in regulating mitochondrial damage and mitophagy in our hyperoxia-induced model. Our data demonstrated that the miR-21-5p could significantly inhibit mitochondrial damage and mitophagy in AEC-II cells to protect HALI through directly binding to PGAM5 3′UTR.

## 2. Materials and Methods

### 2.1. Hyperoxia-Induced Acute Lung Injury Model (HALI Model)

Thirty male Sprague-Dawley rats (200-220 g) were obtained from the Model Animal Research Center of Nanjing University. The rats were given free access to standard rodent food and tap water. All animal experiments were approved by the Animal Care and Use Committee of Zunyi Medical University. According to our previous study [16], the rats were randomly divided into three groups. In order to establish the acute lung injury model, rats were exposed to high concentration (≥95%) oxygen at a flow rate of around 5.0 L/min for 24 h or 48 h. The control group was maintained in the normoxic environment.

### 2.2. Histological Analysis

The rats were anesthetized after different treatments. Rat lungs were isolated and immersed in 4% paraformaldehyde for 24 h at 4°C. Lung tissues were then embedded in paraffin and sliced into five-micrometer sections. After dewaxing and hydration, the sections were immersed in hematoxylin for 5 min and then treated with an eosin solution for 1 min. Then, the sections were dried and mounted with neutral gel. Finally, the sections were observed by light microscopy.

### 2.3. Measurement of Cytokines/Chemokines in Bronchoalveolar Lavage (BAL)

After different treatments, the rats were euthanized, and the tracheas were isolated immediately. Then, 5 mL phosphate buffer saline was infused and recollected gently through the trachea (BAL fluid). After centrifuging at 1200 × g for 5 min, the supernatants were used to measure the concentration of malondialdehyde (MDA, Solarbio, Beijing, China) and tumor necrosis factor (TNF-*α*, Abcam, USA) by the commercial kits (Nanjing Jiancheng, China) according to the manufacturer's instruction, respectively [17].

### 2.4. Lung Wet/Dry Weight Ratio

When the rats were euthanized, the lung tissues were isolated from the heart, trachea, and main bronchi. Each lung was wiped dry and weighed and then incubated at 70°C until the weight was not changed. The dry weight was recorded. The ratio was calculated to measure lung edema and water content according to the previous studies [15, 18].

### 2.5. Western Blot Analysis

The lung tissues were isolated from the mice with different treatments. The cells were collected after normoxia or hyperoxia exposure. Total proteins were then extracted using a Total Protein Extraction Kit (Synthgene Biotech, Nanjing, China) following the manufacturer's protocol. The protein concentration was measured by a BCA Protein Assay Kit (Synthgene Biotech, Nanjing, China) following the manufacturer's protocol. Equal protein of each group (30 *μ*g per lane) was separated by 10% SDS-PAGE and transferred onto polyvinylidene fluoride membranes. After blocking with 5% nonfat milk solution for 1 h at room temperature, the membranes were incubated with primary antibodies against light chain 3 (LC3) (1 : 1000, Abcam, Shanghai, China), p62 (1 : 1000, Abcam, Shanghai, China), Beclin1 (1 : 1000, Abcam, Shanghai, China), phosphoglycerate mutase family member 5 (PGAM5, 1 : 1000, Abcam, Shanghai, China), TIM23 (1 : 1000, Abcam, Shanghai, China), TOM20 (1 : 1000, Abcam, Shanghai, China), PINK1 (1 : 1000, Abcam, Shanghai, China), Parkin (1 : 1000, Abcam, Shanghai, China), COX IV (1 : 10000, Abcam, Shanghai, China), and GAPDH (1 : 10000, Abcam, Shanghai, China) overnight at 4°C. Then, membranes were incubated with HRP-conjugated secondary antibody (anti-mouse IgG or anti-rabbit IgG, 1 : 5000, Abcam, Shanghai, China) for 2 h at room temperature. The pictures were detected by a HiSignal™ ECL WB Detection Kit (Synthgene Biotech, Nanjing, China) following the manufacturer's protocol and quantified with ImageJ software.

### 2.6. Cell Culture

The isolation of AEC-II cells was performed based on previous studies [16, 17, 19]. In brief, rat lungs were obtained and washed by PBS several times to remove the blood and leukocytes. The tissues were then cut into small pieces and digested by 0.25% trypsin for 25 min at 37°C. After filtering and centrifugation at 1000 × g for 5 min, the cell pellets were collected. Cells were then digested by collagenase for 15 min at 37°C followed by several washes. In order to remove the fibroblasts, cell pellets were placed on a Petri flask overnight for differential adherence. The purity and survival rates of the cells were determined to be >90%. For the cell experiments, 500 *μ*M H_2_O_2_ was used to simulate the hyperoxic condition [20]. The control group was maintained in the normoxic condition.

### 2.7. RNA Extraction and Reverse Transcription-Quantitative Polymerase Chain Reaction (RT-qPCR)

The lung tissues were isolated from the mice with different treatments. The cells were collected after normoxia or hyperoxia exposure. For the detection of miRNA expression, total RNA was extracted using a Total RNA Extraction Kit (Synthgene Biotech, Nanjing, China) following the manufacturer's protocol. RNA quantity and purity were measured by a NanoDrop spectrophotometer (Thermo Fisher, Wilmington, DE, USA). The cDNA was synthesized using a Strand cDNA Synthesis kit (Takara Biotech, Dalian, China) following the manufacturer's protocol. The protocol was showed as follows: 42°C for 60 min, 70°C for 15 min, and chilling at 4°C. qRT-PCR was then performed using TaqMan™ Fast Advanced Master Mix (Thermo Fisher, Wilmington, DE, USA) with the following procedure: 95°C for 5 min, 95°C for 15 s and extension for 1 min at 60°C for 40 cycles, and 60°C for 5 min. U6 and GAPDH were used as an internal control. The data were normalized by the U6 or GAPDH with the 2^-*ΔΔ*CT^ method [21]. The primers are the following: miR-21-5p: forward 5′-GTCAATAGCTTATCAGACTGA-3′ and reverse 5′-GTTGGCTCTGGTGCAGGGTCCGAGGTATTCGCA-3′; U6: forward 5′-CTCGCTTCGGCAGCACACG-3′ and reverse 5′-AACGCTTCACGAATTTGCGT-3′; PGAM5: forward 5′-GTGCCACTGGATTAGGGCAA-3′ and reverse 5′-GGGACTTCTCTGACCAGGCT-3′; and GAPDH: forward 5′-CAGGACCTCACTCATTGCCC-3′ and reverse 5′-GACGGACACATTGGGGGTAG-3′.

### 2.8. Measurement of Mitochondrial Membrane Potential

The mitochondrial membrane potential (*Δψ*m) was monitored using a J Mitochondrial membrane potential assay kit with JC-1 (Beyotime, Shanghai, China) according to the manufacturer's instructions. Briefly, after treatment, the AEC-II cells were washed twice with HBSS (Sigma) and incubated in the dark with JC-1 (7.5 *μ*M; 30 minutes at 37°C). Then, the cells were washed with JC-1 wash buffer. Fluorescence images were captured by confocal microscopy [18].

### 2.9. Detection of Mitophagy

For the mitophagy assay, AEC-II cells were seeded onto glass coverslips and transfected with GFP-LC3 plasmid using the HiTrans™ LipoPlus Reagent (Synthgene Biotech, Nanjing, China). After 48 h, cells were subjected to hyperoxia exposure (500 *μ*M H_2_O_2_) with different treatments. Cells were then washed twice with PBS and incubated with 100 nM MitoTracker Red (Molecular Probes, M7512) at 37°C for 15 minutes. The cells were then fixed with 4% paraformaldehyde for 10 min at room temperature, and images were observed by a confocal microscope (Fluoview FV1000, Olympus). The experiments were repeated at least three times independently.

### 2.10. Cell Transfection

AEC-II cells were seeded on a 6-well plate and transfected with 500 nM miR-21-5p mimic or mimic control (mimic-NC), 0.5 *μ*g PGAM5 overexpression plasmid (PGAM5 OE), or control plasmid using a Lipofectamine™ 3000 reagent (Thermo Fisher Scientific, Inc.) following the manufacturer's protocol. The miR-21-5p mimic and related control, PGAM5 overexpression plasmid (PGAM5 OE), and control plasmid were purchased from Synthgene (Nanjing, China). After 48 h, the cells were collected for further experiments. For one group, the cells were cotransfected with PGAM5 OE (0.5 *μ*g) and miR-21-5p mimics (100 pmol) using the same reagent.

### 2.11. Luciferase Reporter Assay

A fragment of PGAM5 3′UTR, including the miR-21-5p binding sites, was predicted via TargetScan 7.2 (http://www.targetscan.org/). The luciferase reporter plasmids of pMIR-PGAM5-3′UTR-WT and pMIR-PGAM5-3′UTR-MUT were purchased from Synthgene (Nanjing, China). 10^6^ AEC-II cells were cotransfected with the plasmids (0.5 *μ*g) and miR-21-5p mimics (100 pmol), as well as the mimic control using HiTrans™ LipoPlus Reagent (Synthgene Biotech, Nanjing, China). The luciferase intensity was measured after 48 h using the Dual-Glo Luciferase Assay System (Promega, Shanghai, China) following the manufacturer's protocol. The data were normalized by renilla signals. The experiments were repeated at least three times independently.

### 2.12. Statistical Analysis

All data in this study were shown as the mean ± Standard Error of Mean (SEM). Student's *t*-test was performed to determine the significance between two groups, and one-way or two-way ANOVA with Bonferroni post hoc tests for multiple groups. *P* value < 0.05 was considered statistically significant.

## 3. Results

### 3.1. Hyperoxia Activated Mitophagy and Induced Mitochondrial Dysfunction during HALI In Vivo

**Figure 1:**
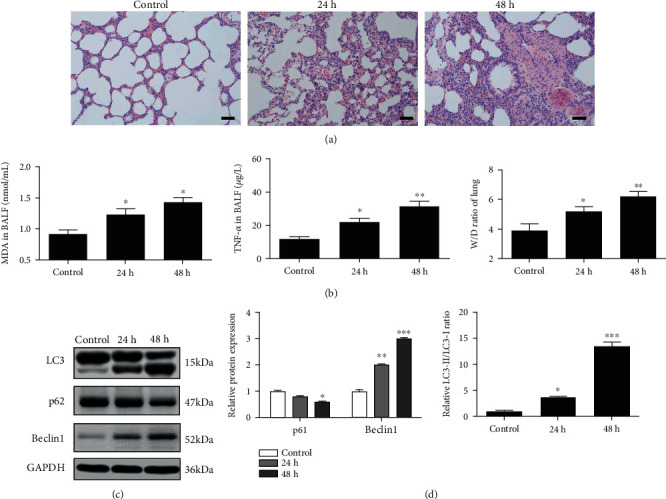
Hyperoxia exposure induced mitophagy during HALI formation. (a) Lung tissues of rats were collected and subjected to hematoxylin and eosin staining. The images were observed under light microscopy (magnification, ×200). Images showed representative examples of three independent experiments. Bar = 20 *μ*m. (b) After different treatments, the W/D ratio of the lung tissues was detected; serum TNF-*α* and MDA levels were determined using ELISA. (c) Representative blots and densitometric analysis were shown for the expression level of LC3, PINK1, Parkin, and COX IV in rat lung tissues. (d) Representative blots and densitometric analysis were shown for the expression level of TIM23, TOM20, p62, Beclin1, and GAPDH in rat lung tissues. *N* = 6. Data were presented as the mean ± SEM. ^∗^*P* < 0.05, ^∗∗^*P* < 0.01, and ^∗∗∗^*P* < 0.001.

According to our previous study, we successfully established a lung injury model through hyperoxia administration [15]. Rats exposed to high oxygen showed diffuse pathological changes, including alveolar congestion and inflammatory cell infiltration. Moreover, the degree of injury was aggravated in a time-dependent manner (Figure 1(a)). A significant time-dependent increase had shown in the injury characteristics, including the W/D ratio of lung tissues and TNF-*α* in the BALF. Compared with the control group, the level of MDA had increased to 1.32 ± 0.13 nmol/mL after hyperoxia exposure for 24 h (*P* < 0.05) and further reached 1.44 ± 0.15 nmol/mL at 48 h after hyperoxia administration (Figure 1(b)).

**Figure 2:**
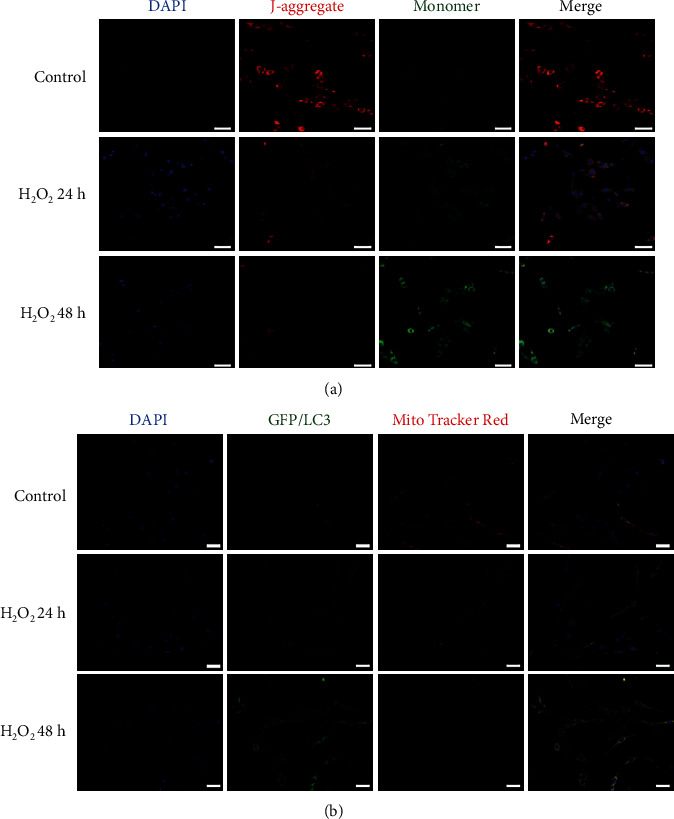
Hyperoxia exposure induced mitochondrial dysfunction during HALI formation. AEC-II cells were isolated from the rats and treated with 500 *μ*M H_2_O_2_ for 24 or 48 h. (a) Representative confocal microscopy images of JC-1 staining and (b) MitoTracker Red/GFP-LC3 staining of the cells with different treatments were performed. Images showed representative examples from three independent experiments. Bar = 20 *μ*m.

To determine the effect of hyperoxia on mitophagy in vivo, we first assessed the changes in mitophagy protein markers using western blot. The result showed that the expression of mitophagy marker PINK1 and Parkin was significantly elevated along with the prolonged hyperoxia exposure. As a critical pathway in mitophagy regulation, our results indicated that the mitophagy occurred during HALI. Besides, hyperoxia exposure significantly regulated the expression of autophagy-related proteins, with an 8-fold increase of the LC3-II/LC3-I ratio and 5-fold upregulation of Beclin1 expression, as well as a significant decline of the p62 level, in the lung tissue of the hyperoxia 48 h group as compared to the control group. In addition, we found that the expression of mitophagy markers like TIM23 and TOM20 was dropped as compared to that in the control group, suggesting that the mitochondrial content was lost under the hyperoxic condition (Figures 1(c) and 1(d)). We then checked the mitochondrial function by a fluorescence assay. AEC-II cells were isolated from lung tissues of normal rats and treated in vitro with H_2_O_2_ exposure for 24 h or 48 h. As shown in Figure 2(a), compared with the control group, the red signal of J-aggregate was time-dependently reduced, while the green signal of monomer was inclined in the H_2_O_2_-treated groups.

Next, we sought to identify whether there was a link between severe mitochondrial damage and the formation of the autophagosome in AEC-II cells. Cells were transfected with both MitoTracker Red and GFP-LC3, following exposure to H_2_O_2_ for 24 h or 48 h. As shown in Figure 2(b), with time increased, the presence of LC3 puncta was raised and showed a significant overlap with mitochondria. All of these data showed that mitochondrial damage has been aggravated under a long-time hyperoxia administration and thus initiated related mitophagy in the AEC-II cells.

## 4. miR-21-5p Alleviated Mitochondrial Dysfunction and Decreased Mitophagy in AEC-II Cell Model

**Figure 3:**
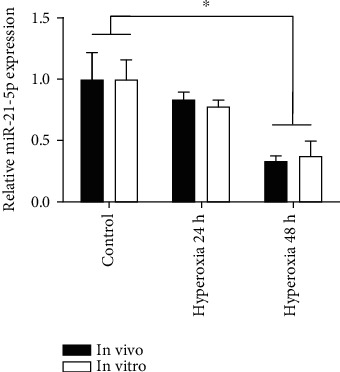
The expression level of miR-21-5p in hyperoxic rat lung tissues and AEC-II cells with different treatments. Data are presented as the mean ± SEM. ^∗^*P* < 0.05*vs.* the control group.

Our previous study has demonstrated that hyperoxia exposure markedly decreased miR-21-5p expression in AEC-II cells. Here, we also confirmed that the expression of miR-21-5p was time-dependently downregulated in the rats subjected to hyperoxia. The level of miR-21-5p was dropped about 2-folds when the rats were maintained in the hyperoxia condition for 48 h (*P* < 0.05). Consistently, we investigated that the miR-21-5p level was also decreased along with the prolongation of stimulation time in vitro. The most significant decrease in it was showed in the 48 h treatment group (*P* < 0.05) (Figure 3).

**Figure 4:**
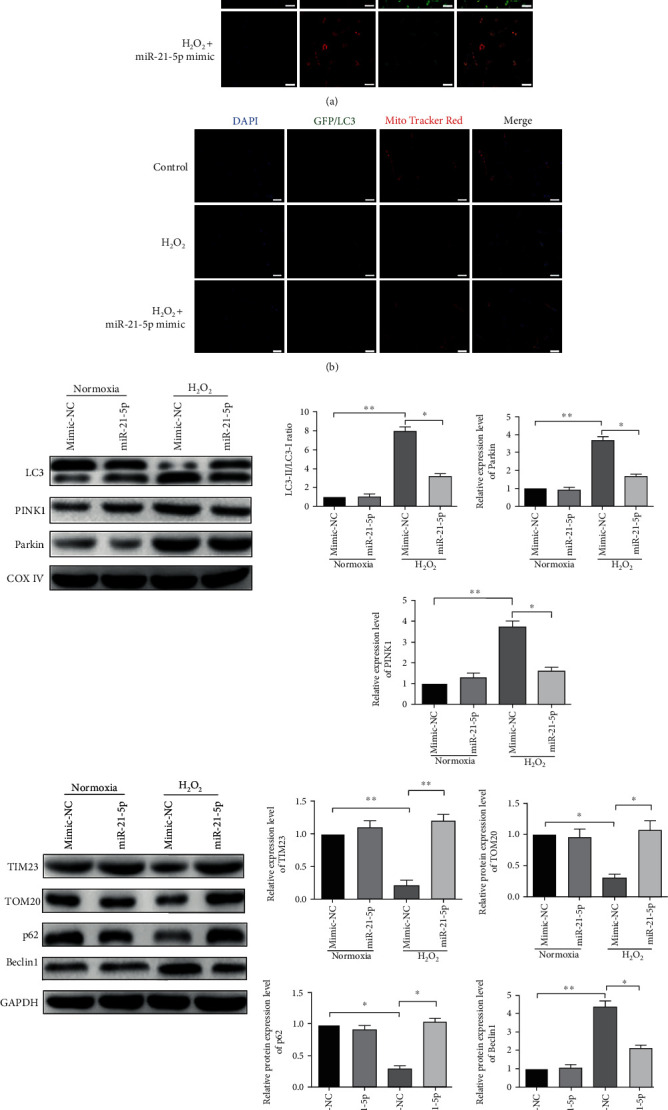
Effect of miR-21-5p on mitochondrial damage and mitophagy of AEC-II cells. (a) Representative confocal microscopy images of JC-1 staining and (b) MitoTracker Red/GFP-LC3 staining of the AEC-II cells with different treatments were performed. Images showed representative examples from three independent experiments. Bar = 20 *μ*m. (c) Representative blots and densitometric analyses were shown for the expression level of LC3, PINK1, Parkin, and COX IV in AEC-II cells after different treatments. (d) Representative blots and densitometric analyses were shown for the expression level of TIM23, TOM20, p62, Beclin1, and GAPDH in AEC-II cells with different treatments. *N* = 6. Data are presented as the mean ± SEM. ^∗^*P* < 0.05, ^∗∗^*P* < 0.01, and ^∗∗∗^*P* < 0.001.

To investigate the role of miR-21-5p in HALI, H_2_O_2_-treated AEC-II cells were transfected with miR-21-5p mimic or mimic-NC. As expected, compared with the control group, the mitochondrial membrane potential level was decreased markedly, while miR-21-5p mimic treatment significantly reversed the reduction of the mitochondrial membrane potential level to repair the mitochondrial dysfunction. Then, we used the fluorescence of mitochondria and lysosome to observe the mitophagy. The overlap of mitochondria and lysosome was induced, comparable to the cells exposed to hyperoxic condition alone (Figures 4(a) and 4(b)).

Accordingly, H_2_O_2_ administration increased the expression level of PINK1 and Parkin, while downregulating the protein level of TIM23 and TOM20. In addition, the LC3-II/LC3-I ratio was significantly increased under the H_2_O_2_ exposure than that in the normoxia+mimic-NC group, which was 39.3 ± 5.32 and 3.22 ± 1.21, respectively (*P* < 0.001). Similarly, a 40-fold upregulation of the Beclin1 expression had been observed in the H_2_O_2_+mimic-NC group (*P* < 0.001). Contrarily, compared with the normoxia+mimic-NC group, there was a marked decline in the p62 expression level of the H_2_O_2_+mimic-NC group (*P* < 0.01). However, the anomalous change of these protein markers had been reversed after miR-21-5p mimic administration (Figure 4(c)). These data offered evidence that miR-21-5p played an important role in mitophagy and mitochondrial function during hyperoxia treatment.

## 5. miR-21-5p Directly Targets PGAM5 in AEC-II Cells

**Figure 5:**
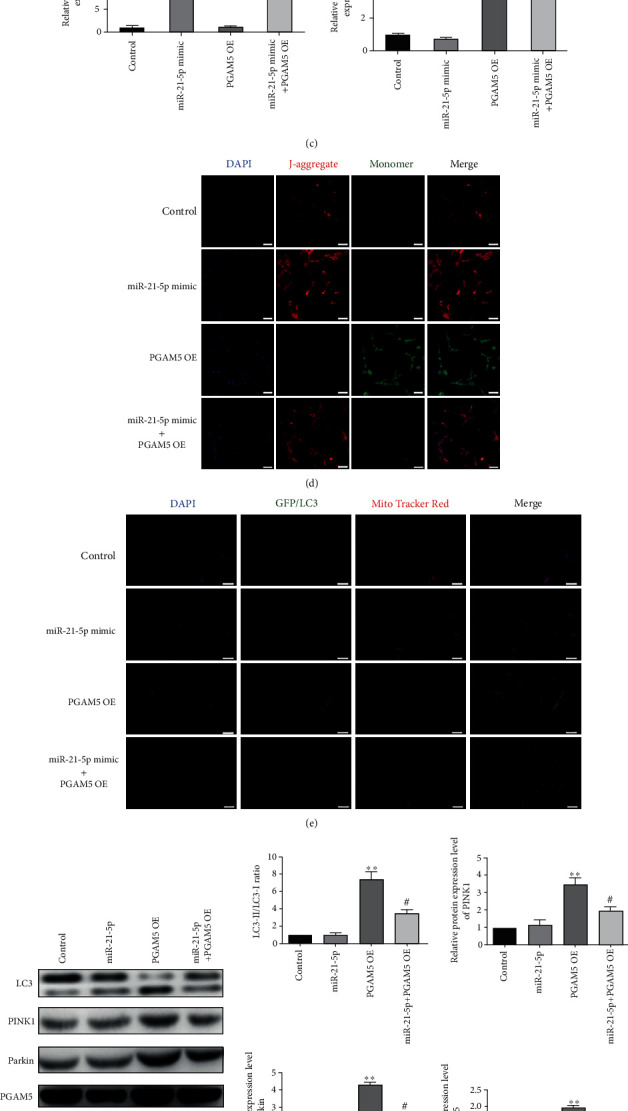
miR-21-5p directly targets PGAM5 to regulate mitophagy in AEC-II cells. (a) Sequence alignment of the PGAM5 3′UTR with miR-21-5p. (b) The activity of luciferase reporters containing wild-type (WT) or mutant (MUT) PGAM5 3′UTR that were treated with miR-21-5p mimics or mimic control (mimic-NC). (c) The transfection efficiency of miR-21-5p mimic and PGAM5 plasmid in AEC-II cells. (d) Representative confocal microscopy images of JC-1 staining and (e) MitoTracker Red/GFP-LC3 staining of the AEC-II cells with different treatments were performed. Images showed representative examples from three independent experiments. Bar = 20 *μ*m. (f) Representative blots and densitometric analyses were shown for the expression of PGAM5, LC3, PINK1, Parkin, and COX IV in AEC-II cells after different treatments. *N* = 6. Data are presented as the mean ± SEM. ^∗^*P* < 0.05, ^∗∗^*P* < 0.01, and ^∗∗∗^*P* < 0.001*vs.* the control group; ^#^*P* < 0.05, ^##^*P* < 0.01, and ^###^*P* < 0.001*vs.* the PGAM5 OE group.

To identify the target of miR-21-5p, the TargetScan database (http://www.targetscan.org) was used to search the potential targets. Among them, PGAM5 was the candidate gene with the highest score. The binding sites between miR-21-5p and PGAM5 are presented in Figure 5(a). In order to determine the mechanism of miR-21-5p, the wild-type or mutant PGAM5 3′UTR were designed and constructed in the downstream of a luciferase reporter gene. The luciferase intensity was reduced obviously by a miR-21-5p mimic in the wild-type group rather than the mutant one (*P* < 0.05), indicating that miR-21-5p specially banded to the 3′UTR of PGAM5-WT and regulated the PGAM5 expression negatively (Figure 5(b)).

## 6. Hyperoxia Activated Mitophagy and Induced Mitochondrial Dysfunction via miR-21-5p/PGAM5 Axis

To further confirm the function of the miR-21-5p/PGAM5 axis, we transferred PGAM5 plasmid into AEC-II cells under normoxic condition and treated them using miR-21-5p mimic. As shown in Figure 5(c), a 16-fold increase and 6-fold upregulation were observed in the cells after miR-21-5p mimic or PGAM5 plasmid transfection, respectively. Besides, the overexpression of PGAM5 could highly decline the mitochondrial membrane potential level and increase the expression level of LC3 in mitochondria. With miR-21-5p treatment, these phenomena had been reversed (Figures 5(d) and 5(e)). Consistently, as shown in Figure 5(f), miR-21-5p treatment notably decreased the protein level of PGAM5 as compared to the control group (*P* < 0.05). Moreover, the western blot results demonstrated that the overexpression of PGAM5 in the cells might dramatically upregulate the LC3-II/LC-I ratio and the expression level of PINK1 and Parkin (*P* < 0.01). However, when the cells were transfected with miR-21-5p mimic and PGAM5 plasmid, the protein levels of these markers were decreased, which suggested that the phenomena of mitophagy were abolished. These experiments revealed that miR-21-5p might suppress mitophagy through binding to PGAM5 3′UTR.

## 7. Discussion

Exposure to the high concentrations of oxygen causes a massive increase in reactive oxygen metabolites, leading to severe inflammation and cell apoptosis in the lungs, which limits the application of oxygen [22]. In this study, we first successfully established a rat model of HALI. After incubation with a high level of oxygen, the rats showed significant lung injury in a time-dependent manner. We further found that the expression level of LC3, p62, and Beclin1 was regulated by hyperoxia exposure, indicating that autophagy had been activated during HALI. Previous studies showed that autophagy is an evolutionarily conserved physiological process, which upregulated rapidly under various stress conditions, including oxidative stress and hyperoxia [23, 24].

As a kind of special autophagy, mitophagy acts as a selective scavenger of mitochondria in physiological and pathological conditions. Numerous studies have proved that the PINK1-Parkin pathway might be activated during the process of mitophagy, accompanied by increased LC3 maturation and declined expression of mitophagy markers such as TIM23 and TOM20, which serve as the biomarker for mitophagy identification [25, 26]. Nowadays, emerging evidence shows that mitophagy may subsequently attenuate apoptosis to maintain normal tissue homeostasis [27]. However, in some specific cellular settings, mitophagy may lead to cell death [28, 29]. In this work, after H_2_O_2_ treatment, a time-dependent mitochondrial dysfunction had been performed with the loss of the mitochondrial membrane potential level. Consistently, an increasing number of mitochondria surrounded by lysosomes have been detected with 24 h and 48 h treatment, suggesting mitochondrial mass loss and mitochondrial dysfunction occurrence during HALI. Our findings also demonstrated that H_2_O_2_ exposure could activate mitophagy as evidenced by the increased LC3-II/LC3-I ratio and activated PINK-Parkin pathway in AEC-II cells.

MicroRNAs (miRNAs) can negatively regulate gene expression at the posttranscriptional level [30]. Many researchers have demonstrated that overexpression of miR-21-5p in some types of cancer cells has been proved to involve in cellular proliferation and apoptosis [31]. For example, previous studies reported that miR-21-5p inhibits apoptosis in a breast cancer mouse model and gemcitabine-induced cells [32, 33]. Interestingly, in this work, we found that miR-21-5p was expressed both in rat lungs and AEC-II cells. The level of it was significantly decreased after long-time hyperoxia treatment. Then, using genetic gain-of-function strategies, we revealed a promising causal role of increased miR-21-5p, protecting against mitochondrial damage and related mitophagy in AEC-II cells.

To further confirm the target of miR-21-5p, the TargetScan database (http://www.targetscan.org) was used. We found that PGAM5 was a possible target of miR-21-5p. As a critical driver of mitochondrial dysfunction, PGAM5 played an essential role in the intrinsic apoptosis and necrosis pathway [34, 35]. In our model, we identified that overexpression of PGAM5 could highly reduce the mitochondrial membrane potential level and promote mitophagy. miR-21-5p negatively regulated expression of PGAM5 through directly banding the 3′UTR of PGAM5 mRNA. Our data thus revealed the role of miR-21-5p and PGAM5, which was believed to be necessary for the regulation of mitochondrial function and the related HALI. All these results indicated that miR-21-5p inhibited hyperoxia-induced HALI, through directly suppressing PGAM5 function in AEC-II cells.

In summary, this study identified that mitophagy was involved in the pathological process of hyperoxia-induced acute lung injury. miR-21-5p contributed to protecting HALI by influencing mitophagy through regulation of the *PGAM5 gene.*

## Data Availability

The data used to support the findings of this study are available from the corresponding author upon request.
